# Supplementary material for: Tximeta: Reference sequence checksums for provenance identification in RNA-seq
Source: PLoS Comput Biol. 2020 Feb 25;16(2):e1007664. doi: 10.1371/journal.pcbi.1007664 (PMC7059966; doi:10.1371/journal.pcbi.1007664)
Supplement: Attachment — Submitted filename: tximeta - response to reviewers.pdf [file pcbi.1007664.s001.pdf]

January 3, 2020

Response to reviewers for manuscript:

"Tximeta: reference sequence checksums for provenance identification in RNA-seq"

We thank the reviewers for their helpful comments which we feel have greatly improved our manuscript. In summary, we have added a new table and a subsection to the Design and Implementation section, both of which compare the proposed software, tximeta, with other related software, and discuss the novel contributions. We have also added the two features suggested by Reviewer #2 to tximeta, as well as a feature to make it easier to use tximeta with quantification software other than Salmon / alevin, as suggested by Reviewer #1. We have revised the text to clarify all of the sections indicated by the reviewers.

We were glad to see that both reviewers agree with the impact of the software described in the submission, that tximeta is "potentially highly useful for improving data provenance and reproducibility" and that it "facilitates RNA-seq data analysis considerably and helps to avoid the common pitfall of using incorrect annotations on quantified transcript tables."

We respond to each of the reviewers comments point by point below.

Reviewer #1:

Major comments:

- As the idea itself is relatively simple, I think it should ideally be presented in a shorter form than the current nine pages. For instance, the Results section is, in my opinion, unnecessarily detailed in its present form, with excerpts and details that would seem more suited for supplementary material or a use case on a supporting web page. Examples of this include the excerpts in line 216-220 [the output of tximeta] and part of the details given in line 232-237 [what metadata columns are available for various references]. If possible, I think it would also be advantageous to get to the point a bit quicker in the introduction and cut it down from the current two pages to maybe half. There are also aspects of the work that I currently found a bit unclear, so I would encourage to also consider my comments below in light of compactness of presentation.

We agree with the reviewer that the output of tximeta can be omitted to save space, and have removed these chunks of output.

However we feel the description of the specific information returned by tximeta (e.g. chromosome names, start and end positions, gene ID, biotype, etc.) in the first submission's lines 232-237 (now lines 275-280) should be kept as it provides useful information about what information is provided by tximeta.

The length of the Results section is within the bounds of other Software category papers published in PLOS Computational Biology, and we feel contains the necessary information to understand what the software accomplishes when it is run. In general, the current 11 pages of text from title page to references is in the typical range of Software articles published in this journal. The page length breakdown currently is ~2 pages Introduction, ~3 pages Design and Implementation, ~2.5 pages Results, 1 page Availability and Future Directions.

- The introduction gave a very good overview of existing relevant solutions. However, I believe it could be clearer in describing what it conceptually shares with related approaches and what is unique - I found the introduction to be a bit vague in terms of describing and contrasting tximeta to the described existing solutions. A more systematic categorization of approaches and their features would probably be useful, including more explicitly providing a rationale for the current work by discussing limitations of current approaches. Also a table or figure would probably help make it even clearer. Also, is the post hoc possibilities mostly unique to this tool? As the introduction is quite long already, I would suggest to ensure that such a clarification does not increase the length.

We thank the reviewer for this suggestion and have added Table 1 describing the features of various approaches, and limitations of other current approaches, and a new subsection "Comparison to related software" (lines 201-233) within the Design and Implementations section.

Yes, the unique aspect of tximeta is post hoc identification of reference provenance, and we have now clarified this in the text in a number of places (lines 66, 95, 100 in the Introduction).

- Furthermore, it might be useful to mention the importance and the implications of differences between solutions already in the introduction. An example is that "Tximeta is similar in implementation to the CRAM format in the use of hashed checksums, but identifies the transcript sequences used during sample quantification rather than the genome sequence used during alignment." Here, nothing is stated regarding what are the implications of such a difference.

We have clarified now that CRAM and refget are designed for identification of chromosomes by their sequence, and so are not directly comparable to tximeta, which identifies reference transcript sets (line 49). Furthermore in the Discussion, we have added lines 375-380 which

discuss that a software similar to tximeta for genome-aligned reads would be future work, because tximeta is currently designed for RNA-seq and for transcript-level quantification data import.

- I would have appreciated a clearer presentation of how tximeta fits in as part of an overall reproducible analysis - what comes before and after the use of tximeta. Again, perhaps at least partly in the form of a figure.

We feel that Figure 1 shows well what comes before tximeta (quantification of RNA-seq reads), and what tximeta produces as output. We have now included in the caption that downstream statistical analysis proceeds on the SummarizedExperiment object produced by tximeta. We have also clarified in a number of places that downstream statistical analysis follows after tximeta (lines 165, 268).

- I would have expected to see a brief discussion of potential challenges due to the current limitation to Salmon? How much does this limit current usefulness in the author's view? Would it be problematic to have different provenance schemes for different aligners? And how does this issue relate to that the CRAM format refer to genome sequence.

This is a good point to increase the modularity of the pipeline. We have now modified the tximeta software package in the development branch so that it can be used with any transcriptome-based quantification tool, as long as the tool is wrapped in a workflow that computes the transcript sequence hash value and writes this value to a metadata JSON file in the sample output directory (lines 112-114, 386-388). We have updated the software documentation (in the development branch) with details on how to use tximeta with other quantification tools.

The hash value is required for tximeta to perform its metadata gathering tasks, and currently Salmon is the only software that, when it is run, computes a hash value of the reference sequences and passes this metadata into the sample output. The description of this paradigm is one of the main contributions of the current submission, we believe, and hope that the practice is adopted by other tool developers. However, now thanks to the modifications to the software, any quantification tool could be used with tximeta in combination with a workflow that also involves writing out the hash value of the reference sequences.

With respect to operations on genome-aligned reads and the CRAM format, we now compare to aligned read counting tools in Table 1, and in lines 216-226 in the “Comparison to related software” subsection. We also discuss the limitation that tximeta is only designed for transcript-level quantification data import in lines 375-380 of the Discussion, and not for operations such as genome-aligned read counting in genomic bins.

Given that transcript-based quantification (as provided by Salmon / alevin, kallisto, RSEM, etc.) is a popular choice for RNA-seq gene- and transcript-level analysis, we do not see this as a major limitation, though similar functionality to tximeta for genome-aligned read operations is an interesting direction of future work for our group.

Minor comments:

- The terms hash and checksum seem to be used interchangeably. Are they used in the same or slightly different meanings?

Essentially these are used with the same meaning. A specific hash function produces a hash value. The hash value may also be called a “checksum” or “digest”. A table linking input values (sometimes called keys) to hash values is often called a hash table.

We have used “checksum” in the title and “hashed checksum” throughout the text as more users may be familiar with the concept and word “checksum” than a “hash value” or “digest”. We have attempted to align our terminology with GA4GH and the CRAM/refget developers, which also refer to the values as “checksums”. In the manuscript, we do not ever refer to a “hash” alone but only to “hashed checksums” or a “hash table”.

- Might be useful to separate a bit clearer what is brought by tximeta itself versus what is mostly carried over from underlying tools.

See replies above with respect to Table 1 and new subsection “Comparison to related software”.

- The cover letter claim novelty, while the manuscript itself does not do so explicitly. Related to the point above regarding how it compares to existing approaches, I would encourage to discuss/argue for novelty also in the manuscript.

We feel the revised manuscript sufficiently now fully addresses the novelty of tximeta (the ability for post hoc identification of reference sequence provenance) and comparison to existing software. The only other software for RNA-seq data import which can provide post hoc identification is ARMOR, which wraps tximeta for this functionality. We have clarified this in the revised text.

Reviewer #2:

1) For cases in which alignment was performed against transcriptome sequences from Ensembl, the pre-compiled EnsDb annotation database for the corresponding Ensembl version which is available in Bioconductor's AnnotationHub should be used instead of creating such a resource on-the-fly from Ensembl's GTF/GFF3 files. Pre-build EnsDb databases are available for all species in Ensembl and provide additional annotations such as mappings to protein identifiers, NCBI Entrezgene identifiers or, more recent databases, even the G-C nucleotide content of each transcript.

We thank the reviewer for this suggestion, and have added functionality in tximeta version 1.5.8 (development branch). Tximeta will now first check if an EnsDb database exists on AnnotationHub, and if so, it will download and make use of this pre-parsed file instead of downloading and parsing the GTF file. This avoids unnecessary computation and integrates tximeta with the existing AnnotationHub infrastructure. We added an argument to control whether or not tximeta should make use of AnnotationHub, with the default setting to perform the AnnotationHub lookup.

This new behavior of tximeta is described at line 152 in the new manuscript.

2) In addition to adding annotations to the transcript table, it might be useful to have a function that returns the actual TxDb or EnsDb database from which these annotations were taken. This would allow users to extract additional information for the transcripts such as the number of exons of a transcript or to even use the additional functionality of these annotation resources such as mapping to proteins identifiers, conversion of transcriptome to proteome coordinates.

We thank the reviewer for this suggestion, and have added retrieveDb() as a function to tximeta version 1.5.7 (development branch), and added this to the software vignette. This enables the user to have access to the TxDb or EnsDb that is being used as the backend for annotation tasks, by calling, e.g.:

```
edb <- retrieveDb(se)
```

where ‘se’ is the SummarizedExperiment object created by tximeta.

The new function is described in the vignette, in a section “Retrieve the transcript database” that follows the section showing the SummarizedExperiment output of tximeta(), and in the software package man page under a listing of the main functions of the package.
